# T_FH_ Cells Induced by Vaccination and Following SIV Challenge Support Env-Specific Humoral Immunity in the Rectal-Genital Tract and Circulation of Female Rhesus Macaques

**DOI:** 10.3389/fimmu.2020.608003

**Published:** 2021-01-28

**Authors:** Sabrina Helmold Hait, Christopher James Hogge, Mohammad Arif Rahman, Ruth Hunegnaw, Zuena Mushtaq, Tanya Hoang, Marjorie Robert-Guroff

**Affiliations:** Immune Biology of Retroviral Infection Section, Vaccine Branch, National Cancer Institute, National Institutes of Health, Bethesda, MD, United States

**Keywords:** T_FH_ cells, B cell help, humoral immunity, rhesus macaque, SIV vaccine

## Abstract

T follicular helper (T_FH_) cells are pivotal in lymph node (LN) germinal center (GC) B cell affinity maturation. Circulating CXCR5^+^ CD4^+^ T (cT_FH_) cells have supported memory B cell activation and broadly neutralizing antibodies in HIV controllers. We investigated the contribution of LN SIV-specific T_FH_ and cT_FH_ cells to Env-specific humoral immunity in female rhesus macaques following a mucosal Ad5hr-SIV recombinant priming and SIV gp120 intramuscular boosting vaccine regimen and following SIV vaginal challenge. T_FH_ and B cells were characterized by flow cytometry. B cell help was evaluated in T_FH_-B cell co-cultures and by real-time PCR. Vaccination induced Env-specific T_FH_ and Env-specific memory (ESM) B cells in LNs. LN Env-specific T_FH_ cells post-priming and GC ESM B cells post-boosting correlated with rectal Env-specific IgA titers, and GC B cells at the same timepoints correlated with vaginal Env-specific IgG titers. Vaccination expanded cT_FH_ cell responses, including CD25^+^ Env-specific cT_FH_ cells that correlated negatively with vaginal Env-specific IgG titers but positively with rectal Env-specific IgA titers. Although cT_FH_ cells post-2^nd^ boost positively correlated with viral-loads following SIV challenge, cT_FH_ cells of SIV-infected and protected macaques supported maturation of circulating B cells into plasma cells and IgA release in co-culture. Additionally, cT_FH_ cells of naïve macaques promoted upregulation of genes associated with B cell proliferation, BCR engagement, plasma cell maturation, and antibody production, highlighting the role of cT_FH_ cells in blood B cell maturation. Vaccine-induced LN T_FH_ and GC B cells supported anti-viral mucosal immunity while cT_FH_ cells provided B cell help in the periphery during immunization and after SIV challenge. Induction of T_FH_ responses in blood and secondary lymphoid organs is likely desirable for protective efficacy of HIV vaccines.

## Introduction

The development of a safe and effective HIV-1 vaccine remains a critically important global health priority. To date HIV-1 vaccine candidates aimed at eliciting cellular and/or humoral responses have failed to provide significant protection (1). Nevertheless, the RV144 HIV-1 efficacy trial in Thailand showed a modest efficacy of 31.2% (2). Immunological correlates from the RV144 regimen attributed protection against HIV-1 infection to antibodies against the V1-V2 region of the HIV-1 envelope and Env-specific non-neutralizing antibodies with functional activity (3, 4).

Antibody production during vaccination and infection is shaped by T follicular helper (T_FH_) cells, which are a subset of CD4^+^ T cells specialized in promoting B cell expansion and maturation in B cell follicles and germinal centers (GCs) of secondary lymphoid organs (5). T_FH_ cells are characterized by CXCR5 and PD-1 expression (6, 7) and are essential cellular subsets that participate in generation of antigen-specific long-lived plasma cells (PCs) and memory B cells through affinity selection of B cells undergoing somatic hypermutation. Several studies have shown that T_FH_ cells play a crucial role in development of broadly neutralizing antibodies (bNAbs) in HIV infected patients (8–12). Moreover, T_FH_ cell expansion in lymph nodes (LNs) of rhesus macaques has been associated with development of bNAbs against the HIV envelope during SHIV infection (13).

In blood, CXCR5^+^ CD4^+^ T cells, a cellular subset with memory phenotype reported to share functional properties with LN T_FH_ cells, supported T cell-dependent B cell maturation and antibody production *in vitro* (14–16). This peripheral subpopulation exhibited a similar transcriptional profile as GC T_FH_ cells (14), and therefore was identified as circulating T follicular helper (cT_FH_) cells (17–19). HIV-specific cT_FH_ cells were found to be increased in the blood of RV144 vaccine recipients, and these cells have been associated with breadth of NAbs in HIV infected patients, suggesting a role in HIV protection (12, 17, 20, 21). Expansion of HIV-specific memory cT_FH_ cells has also been associated with development of bNAbs in HIV-infected individuals (20, 22, 23), supporting the role of cT_FH_ cells in development of humoral responses against HIV. Expansion of HIV-specific cT_FH_ cells seen in HIV elite controllers suggested a contribution to HIV-specific IgG responses and preservation of HIV-specific memory B cell responses in the circulation (24).

Development of vaccine-induced HIV-specific humoral responses is highly dependent on selection of the HIV-specific B cell repertoire, a process that requires integral participation of HIV-specific T_FH_ cells (25). Induction of Env-specific T_FH_ cell responses by immunization strategies would provide important signals for elicitation of Env-specific antibody responses. Although T_FH_ cells have been intensively investigated during HIV and SIV infection, less is known about T_FH_ cell responses during HIV/SIV immunization and how these T_FH_ responses contribute to protective humoral immunity. In the rhesus macaque SIV/SHIV models, T_FH_ cells have been shown to be induced by vaccination (26, 27) and suggested to play a role in protection against viral infection (28, 29). However, vaccine-induced T_FH_ cells have also been correlated with higher acute viral loads following SIV challenge (18). We recently reported that early induction of T_FH_ cells in GCs of immunized rhesus macaques was important for robust GC maturation associated with viremia control following SIV infection (30). Here, using female rhesus macaques immunized mucosally with replicating Adenovirus type 5 host range mutant (Ad5hr)-SIV recombinants followed by intramuscular gp120 protein boosting (31), we expanded our investigations of T_FH_ cells and their role in development of SIV-specific humoral responses in different mucosal and systemic tissue compartments. Although Ad5 is no longer a viable HIV vaccine candidate in human studies due to previous failures in clinical trials, several other replicating Ad-vectored approaches are being explored (29, 32–34). For investigation of replicating Ad vaccines using the SIV/rhesus macaque model, we have used the Ad5hr vector because it exhibits persistent replication in rhesus macaques cells resulting in provision of long-lasting immune responses (35–38). In this study we performed comprehensive correlation analyses between T_FH_ subsets and antibody responses which indicated that early development of vaccine-induced SIV-specific T_FH_ subpopulations contributed to Env-specific humoral responses in the periphery and female rectal-genital tract, a critical site of HIV/SIV exposure. In addition to analysis of GC T_FH_ cell subsets, we conducted phenotypic and functional characterization of absolute and SIV-specific T_FH_ cells in peripheral blood, allowing us to explore their capacity to provide help to peripheral antibody secreting B cells. Taken together our results provide evidence that future prophylactic HIV vaccines should aim for efficient induction of Env-specific T_FH_ cells in both secondary lymphoid organs and the periphery in order to support development of highly specific anti-HIV Env antibodies.

## Materials and Methods

### Study Animals and Immunization

As previously reported (31) female rhesus macaques were immunized at week 0 (intranasally and orally) and week 13 (intratracheally) with replicating Ad5hr recombinants expressing SIV_smH4_ *env/rev*, SIV_239_ *gag*, and SIV_239_ *nef* (*n* = 38, Ad-SIV) at a dose of 5 × 10^8^ plaque forming units/recombinant/route/macaque or with Ad5hr empty vector (*n* = 22, Ad-Empty) at a dose equivalent to the Ad-SIV recombinants administered. At weeks 26 and 38, recombinant SIV_M766_ and SIV_CG7V_ gp120 protein boosts (200 μg each protein/dose/macaque) in alum hydroxide were given to the Ad-SIV group (n = 38) while alum only was given to the Ad-Empty group (n = 22). The 2 ml boosts were administered, 1 ml to both the left and right inner thighs, on weeks 26 and 38, respectively. Animals were challenged intravaginally beginning at week 45 with up to 15 repeated weekly low-doses (800 median tissue culture infectious doses) of SIV_mac251_. Twenty of the Ad-SIV and 12 of the Ad-Empty macaques were administered a 0.8% formulation of SAMT-247 (S-acyl-2-mercaptobenzamide thioester) microbicide gel (39) vaginally 3 h prior to each challenge. The use of the vaginal microbicide together with the Ad-SIV based vaccination regimen aimed to evaluate a potential synergic protective effect of the combination approach as has recently been reported in detail (31). All rhesus macaques were maintained at the NCI animal facility under the guidelines of the Association for the Assessment and Accreditation of Laboratory Animal Care. The protocol (VB-027) and procedures were approved by the NCI Animal Care and Use Committee prior to initiation of the study.

### Sample Collection and Processing

For assessment of vaccine dependent T_FH_ cell and B cell responses, LN biopsies were obtained 4 weeks prior to vaccination, and 3 days and 14 days following the 2nd adeno and 2nd boost immunizations. In order to minimize sequential LN sampling of all macaques, 13 animals had LN biopsies prior to and 3 days following immunizations whereas 10 animals had LN biopsies collected prior to and 14 days following immunizations. Axillary LN biopsies were collected prior to immunization. Inguinal LNs were collected following the second immunization with Ad5hr -SIV replicating vectors (from the left thigh) and following the second immunization with gp120 proteins (from the right thigh). Blood was also collected at the same times as LN biopsies for evaluation of cT_FH_ cell responses. Twenty-one macaques were analyzed for cT_FH_ phenotype and dynamics over the course of immunization: 12 macaques at day 3 following immunizations and nine at day 14 following immunizations.

To evaluate vaccine-induced systemic and mucosal humoral immune responses, bone marrow, vaginal washes and rectal swabs were obtained from all macaques 4 weeks prior to vaccination and 3 weeks following each immunization. Lymphocytes were isolated from LN biopsies as previously described (40) and stored frozen in FBS/10% DMSO solution. Blood and bone marrow samples were centrifuged over Ficoll gradients for isolation of leukocytes, treated with ACK buffer for red blood cell lysis and stored frozen in FBS/10% DMSO as previously described (41).

Vaginal washes were collected with 2 ml PBS and placed in 15 ml conical tubes, centrifuged to pellet vaginal epithelial cells, and the supernatants were stored frozen at -80°C until analyzed. Rectal secretions were collected with polyester-tipped swabs (Becton-Dickson, Cockeysville, MD, USA) and transferred into cryovials containing 0.1% BSA, 0.01% thimerosal and 750 Kallikrein inhibitor units of aprotinin/ml storage solution (all from Sigma Aldrich). Both rectal swab solutions and vaginal washes were used for gp120 specific IgG and IgA detection by ELISA.

### Flow Cytometric Detection of LN T_FH_ Cells, cT_FH_ Cells, and LN B Cells

Env-specific T_FH_ cells in LN GCs and cT_FH_ cells in blood were detected by the Activation Induced Marker (AIM) assay using CD25 and CD134 for identification of antigen-specific T cells as described elsewhere (42). Identification of antigen-specific cT_FH_ used CD25 only. Absolute LN T_FH_ cells were defined as PD-1^high^ CXCR5^+^ CD4^+^ CD3^+^ T cells and GC T_FH_ cells were defined as PD-1^high^ CXCR5^+^ CD4^+^ CD3^+^ T cells as previously reported (43). Absolute cT_FH_ cells were defined as CXCR5^+^ CD4^+^ T cells (18). Briefly, 2x10^6^ LN cells or PBMCs were either stimulated with 1 µg/ml pooled SIV_CG7V_ gp120 peptides consisting of complete sets of 15-mer peptides overlapping by 11 aa (Advanced Bioscience Laboratories Inc, Rockville, MD; ABL), or remained non-stimulated. LN cells or PBMCs stimulated with 1X PMA/Ionomycin cell stimulation cocktail (eBioscience) were used as positive controls. All LN cells received a mixture of 2 µg/ml anti-CD49-d and anti-CD28 as well as APC-eFluor780 anti-CCR7 (**Table 1**) and were incubated for 43 h at 37°C and 5% CO_2_. After incubation, the cells were stained for surface markers (**Table 1**), washed, permeabilized with the Foxp3/transcription factor buffer set (eBioscience) and incubated with intracellular staining (ICS) antibodies (**Table 1**). Finally, cells were resuspended in 2% formaldehyde (2% PFA) fixation solution and maintained at 4°C until acquisition. LN Env-specific AIM^+^ (CD25^+^CD134^+^) cells and Env-specific AIM^+^ cT_FH_ cells (CD25^+^) were calculated by subtracting non-stimulated values from stimulated values. For detection of cT_FH_ cells, the AIM assay was performed using the same protocol as for LN T_FH_ cells, with inclusion of CD14 and CD16 antibodies to the surface staining panel for exclusion of monocytes and macrophages (**Table 1**).

**Table 1:** Antibodies used in flow cytometry assays for identification of T_FH_ cells in LNs, cT_FH_ cells in PBMCs, and B cells in LN and PBMCs.

Assay	Marker	Clone	Fluorochrome	Company	Staining
**AIM assay, LN T_FH_ Cells**	CD49d	R1-2	unconjugated	BD Biosciences	Stimulation
	CD28	CD28.2	unconjugated	BD Biosciences	Stimulation
	CCR7 (CD197)	2D12	PerCP-eFluor780	eBioscience	Stimulation
	PD-1	EH12.2H7	BV605	BioLegend	Surface
	CD25	BC96	PE-Cy5	BioLegend	Surface
	CXCR5	MU5UBEE	PerCP-eFluor710	eBioscience	Surface
	CD134	L106	PE	BD Biosciences	Surface
	CD4	L200	BV711	BD Biosciences	Surface
	Live/Dead	–	Aqua Dye	Invitrogen	Surface
	Ki67	B56	BV786	BD Biosciences	ICS
	CD3	SP34-2	AlexaFluor-700	BD Biosciences	ICS
	Bcl6	K112-91	BV421	BD Biosciences	ICS
**AIM assay, cT_FH_ cells**	CD49d	R1-2	unconjugated	BD Biosciences	Stimulation
	CD28	CD28.2	unconjugated	BD Biosciences	Stimulation
	CCR7 (CD197)	2D12	PerCP-eFluor780	eBioscience	Stimulation
	PD-1	EH12.2H7	BV786	BioLegend	Surface
	CD25	BC96	PE-Cy5	BioLegend	Surface
	CXCR5	MU5UBEE	PerCP-eFluor710	eBioscience	Surface
	CD134	L106	PE	BD Biosciences	Surface
	CD4	L200	BV711	BD Biosciences	Surface
	Live/Dead	–	Aqua Dye	Invitrogen	Surface
	CD14	Tuk4	Qdot605	ThermoFisher	Surface
	CD16	CB16	BV605	eBioscience	Surface
	CD3	SP34-2	AlexaFluor-700	BD Biosciences	ICS
	Bcl6	K112-91	BV421	BD Biosciences	ICS
	Foxp3	206D	Alexa647	BioLegend	ICS
**B cells**	CD4	19thy5D7	unconjugated	NHP reagent resource	Surface
	CD4	OKT4	unconjugated	NHP reagent resource	Surface
	CD2	S5.5	Qdot605	ThermoFisher	Surface
	CD14	Tuk4	Qdot605	ThermoFisher	Surface
	CD20	2H7	BV650	BioLegend	Surface
	CD27	O323	PerCp-Cy5.5	eBioscience	Surface
	IgD	Polyclonal	Texas red	SouthernBiotech	Surface
	CD21	B-ly4	BV711	BD Biosciences	Surface
	CXCR4	12G5	BV785	BioLegend	Surface
	CD95	DX2	Pe-Cy7	BD Biosciences	Surface
	CD19	J3-119	PE-Cy5	Beckman Coulter	Surface
	CD138	DL-101	PE	BioLegend	Surface
	Ki67	B56	Alexa700	BD Biosciences	ICS
	IRF4	IRF4-3E4	FITC	ThermoFisher	ICS
	BCL6	K112-91	APC-Cy7	BD Biosciences	ICS

ICS, Intracellular Staining.

B cells in LN were detected as previously reported (30) with antibodies described in **Table 1**. Envelope protein staining was carried out by incubating cells with biotinylated gp120 proteins (2 µg/sample) at 4°C, following incubation with streptavidin-APC at 4°C. Cells were permeabilized using the Transcription Factor Buffer set (BD Biosciences) according to manufacturer specifications and intracellularly stained using antibodies listed in **Table 1**. Two percent PFA was added and cells were maintained at 4°C until acquisition using an 18 laser LSRII (BD Biosciences) and analyzed with FlowJo 10.2 (FlowJo, Ashland, OR). In order to compare cellular dynamics across the different groups of macaques that had collections at different timepoints, frequencies of LN T_FH_ cell, cT_FH_ cell, and LN B cell subpopulations were normalized to pre-immunization levels and reported as fold-change values or magnitude of response.

### IgA and IgG Binding Titers in Mucosal Secretions and Culture Supernatants

Rectal secretions were thawed and filtered with centrifugal filters (Durapore PVDF 5.0 μm; Merck Millipore, Tullagreen Carrigtwohill, CO) prior to antibody assays. Rectal filtrates and vaginal washes were tested for blood contamination using Chemstrip^®^ 5OB urine test strips (Roche, Indianapolis, IN). Samples in which blood was detected were not assayed. ELISAs for vaginal and rectal secretions were carried out as previously described (31). Standard curves for quantifying total IgG and IgA antibody were generated using standards obtained from the Nonhuman Primate Reagent Resource. Goat anti-monkey HRP conjugates were used as detection antibodies at a 1:10000 dilution. Env-specific IgG and IgA, derived from purified serum IgG and IgA obtained from SIV_mac251_-infected macaques and quantified as reported (44), was used to generate a standard curve for Env-specific IgG and Env-specific IgA. Mucosal antibodies were reported as ng Env-specific IgG or IgA per μg of total IgG or IgA. Additionally, supernatants from cT_FH_ and B cell co-cultures were saved for evaluation of T_FH_-dependent antibody release by ELISA. Total IgG and IgA in supernatants were determined as described above and reported as ng/μl.

### SIV-Specific Antibody Secreting Cells in Bone Marrow and Rectal Biopsies

Both total IgG and IgA and SIV_M766_ and SIV_CG7V_ gp120-specific IgG and IgA antibody secreting cells (ASC) were quantified in bone marrow and rectal mucosa by ELISpot as previously described (45). Env-specific IgA and IgG ASC were standardized to the total number of IgG and IgA ASC and reported as percentage IgA and IgG Env-specific activity relative to the number of total IgG and IgA ASC.

### Isolation of cT_FH_ Cells and B Cells

Viably frozen PBMCs obtained from three macaques prior to immunization, 14 infected macaques 40 weeks post-infection, and nine protected macaques 2 weeks following the last challenge were thawed and used for either negative selection of CD4^+^ T cells (~ 20–40 million cells) or positive selection of B cells (~ 10–20 million cells). CD4^+^ T cells were negatively sorted using the EasySep Rhesus CD4^+^ T cell isolation kit (STEMCELL, Cambridge, MA), stained with anti-CXCR5-PE (Nonhuman Primate Reagent Resource), treated with anti-PE microbeads (Miltenyi Biotech) and positively isolated using a magnetic column. For B cell positive selection, cells were labeled with anti-CD19 magnetic beads (Miltenyi Biotec) and isolated using a magnetic column. To check the purity of the enriched cT_FH_ cells and B cells obtained by this procedure, PBMC obtained from three additional necropsied macaques were stained with Alexa700 anti-CD3 (SP34-2), BV711 anti-CD4 (L200), FITC anti-CD8 (RPA-T8) (BD Bioscience), BV650 anti-CD20 (2H7) (BioLegend), BV605 anti-CD16 (CB16) (eBioscience), and PE-Cy5 anti-CD19 (J3-119) from Beckman Coulter. The cells were acquired on a BD LSRII (BD Biosciences) and analyzed as described above.

### Quantification of Helper Cell Function of cT_FH_ Cells *In Vitro*

Autologous cT_FH_ cells and peripheral B cells were co-cultured using a protocol adapted from a previous report (46). Necropsy samples from 11 chronically infected and 6 protected macaques were included. Of the infected animals, none had received microbicide; eight were vaccinated and three received empty Ad5hr vector at prime and alum only at boost. Protected animals included three vaccinated macaques and three macaques that received empty Ad5hr vector and Alum only. All these protected macaques had received microbicide vaginally approximately 32 weeks prior to necropsy. Autologous cT_FH_ cells and B cells magnetically isolated from PBMCs as described above were plated in a 96-well plate at a density of 200,000 cells/200 µl/well at a 1:1 ratio in R10. B cells were co-cultured with CXCR5^+^ T cells (cT_FH_ cells), CXCR5^neg^ T cells (non-cT_FH_ cells) or cultured alone. Co-culture conditions included a mixture of costimulatory molecules in order to provide adequate activation signals to cT_FH_ cells (46). Cells were stimulated with a cocktail containing Staphylococcal enterotoxin (SEB) at 1 µg/ml, anti-CD3 at 2 µg/ml, anti-CD28 at 2 µg/ml, and anti-CD49d at 2 µg/ml for 7 days, after which cells were harvested, enriched for B cells or T_FH_ cells by magnetic sorting and evaluated by flow cytometry using antibodies described in **Table 1**. The stained cells were acquired using an 18 laser BD LSRII (BD Biosciences) and analyzed with FlowJo 10.2. Co-culture ELISA assays were performed as described above and included supernatants from the 11 chronically infected macaques described above, three protected animals (two vaccinated and one unvaccinated that had received microbicide approximately 32 weeks prior to necropsy), and three naïve macaques.

### B Cell RNA Isolation and cDNA Preparation

B-cells only or B-cells together with cT_FH_ cells were cultured for 6–7 days as described above. Transcriptional analysis of the co-cultured B cells used samples from 3 naïve animals, collected 4 weeks prior to the first prime vaccination. B-cells were collected from individual wells, positively selected using PE-CD19 (Beckman Coulter) and anti-PE magnetic beads (Miltenyi) and lysed by adding RLT buffer. RNA was extracted using the AllPrep DNA/RNA Micro Kit (Qiagen, Valencia, CA, USA). Each homogenized cell lysate was transferred to an AllPrep DNA spin column, and the flow-through was collected for RNA purification. An equal volume of 70% ethanol was added to the flow-through, mixed by pipetting, and transferred to an RNase MinElute spin column. The column flow-through was discarded and the column was washed with RW1 buffer. The spin column was further washed by RPE buffer followed by 80% ethanol. Finally, RNase free water was added to elute the RNA. RNA was measured by NanoDrop and 25 ng RNA was used for cDNA preparation with the RT2 First Strand kit (Qiagen, Valencia, CA, USA). The RNA was treated with genomic DNA elimination mix for 5 min at 42°C followed by 1 min incubation on ice. Equal amounts of reverse-transcription mix and the RNA/genomic DNA elimination mix were gently mixed by pipetting and incubated at 42°C for 15 min. The reaction was stopped by incubating at 95°C for 5 min. The first strand cDNA was diluted with RNase free water and used for Real-Time PCR.

### Real-Time PCR

Relative transcription profiles of 84 cytokine and chemokine genes were determined by qRT-PCR using the RT2Profiler™ PCR Array Rhesus Macaque Cytokines and Chemokines Assay kit (Qiagen, Valencia, CA, USA). In addition, transcription of the housekeeping genes ACTB, B2M, GAPDH, LOC709186, and RPL13A was determined using specific primers in the kit. Real-time PCR reactions were set up in duplicate for each of the cytokines and the housekeeping genes. Amplification conditions were kept identical for all reactions and consisted of the following: 10 min at 95°C, 40 cycles of 15 s at 95°C, and 60 s at 60°C. Reaction samples had a final volume of 25 μl consisting of 12.5 μl of RT2 SYBR Green qPCR Mastermix (Cat No./ID: 330500, Qiagen, Valencia, CA, USA) and 12.5 μl of cDNA. Amplifications were run in an ABI Prism 7500 Sequence Detection System (Applied Biosystems). Expression level differences were assessed using the ΔΔCt method.

### Analysis of Cytokine Producing cT_FH_ Cells Co-Cultured With B Cells

After 7 days of incubation, co-cultured cT_FH_ cells and cognate circulating B cells, both obtained from necropsy samples of six chronically infected macaques (all recipients of mock-vaccine) and six protected animals (three vaccinated and three recipients of mock vaccine) were harvested and cT_FH_ cells were magnetically sorted using the EasySep Rhesus CD4^+^ T cell isolation kit (STEMCELL, Cambridge, MA). Magnetically sorted cT_FH_ cells were treated with BD GolgiPlug and BD GolgiStop for 1 h at 37°C in the presence of 5% CO_2_, and stained with Live/Dead aqua dye followed by surface staining antibodies for cT_FH_ phenotyping (**Table 1**) and permeabilized with the Cytofix/Cytoperm permeabilization kit (Becton-Dickson, Cockeysville, MD, USA). Intracellular staining of IL-21 and BAFF was carried out using the ICS antibodies listed in **Table 1**. Cells were washed and fixed with 2% PFA and saved at 4°C until acquisition in on an18-parameter BD LSRII (BD Biosciences) and analyzed with FlowJo 10.2.

### Statistical Analysis

The Mann–Whitney U test was used for comparisons between different groups of animals, and the Wilcoxon signed rank test was used for paired differences within the same group of animals. Spearman correlations were used when indicated. Statistics were generated using GraphPad Prism.

## Results

### Vaccination Induces Env-Specific T_FH_ Cells in LNs

**Figure 1:**
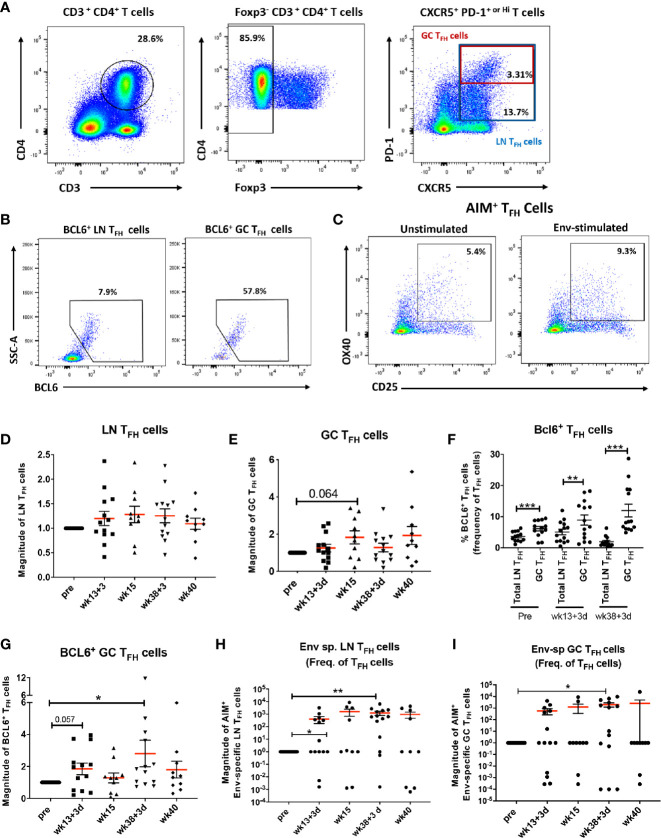
Vaccine-induced T_FH_ cells with GC phenotype and Env-specific T_FH_ cells in LNs. **(A)** Gating strategy used to identify LN and GC-T_FH_ cells in LN of rhesus macaques. **(B)** BCL6^+^ was used to identify T_FH_ cells with GC phenotype. **(C)** The AIM assay was used to identify LN and GC Env-specific T_FH_ cells as described in Methods. Dynamics of **(D)** total LN T_FH_ cells and **(E)** GC-T_FH_ cells assessed at pre, 3 days and 2 weeks post-2^nd^ prime (week 13 + 3 days and week 15), and 3 days and 2 weeks post-2^nd^ boost (week 38 + 3 days and week 40) and represented as magnitude of response. **(F)** Comparison of the frequencies of BCL6 expressing total LN and GC-T_FH_ cells at pre and 3 days following 2^nd^ prime and 2^nd^ boost immunizations. Dynamics of **(G)** BCL6^+^ GC-T_FH_ cells, **(H)** Dynamics of Env-specific LN T_FH_ cells, and **(I)** Env-specific GC-T_FH_ cells over the course of immunization. Statistics were generated using the Wilcoxon signed rank test for animals within the same group or the Mann-Whitney U test for animals from different groups **(D**–**I)**. * indicates p < 0.05, ** indicates p < 0.01, *** indicates p < 0.001.

Initiation of the GC reaction and generation of antigen-specific B cells in secondary lymphoid organs depends on signals provided by T_FH_ cells (47, 48). To determine whether the vaccine regimen elicited LN T_FH_ cell responses in the rhesus macaques, after gating out Foxp3^+^ T cells for exclusion of T follicular regulatory (T_FR_) cells, total LN T_FH_ cells were identified as PD-1^+^ CXCR5^+^ CD4^+^ T cells. PD-1 upregulation is required for positioning T_FH_ cells within GCs (49). GC-T_FH_ cells were identified as PD-1^hi^ CXCR5^+^ CD4^+^ T cells as previously described (30, 43) (**Figure 1A**). The transcription factor BCL6 (B-cell lymphoma protein 6) supports the differentiation of T cells into T_FH_ cells by activating upregulation of PD-1 and defines GC-committed T_FH_ cells (50). BCL6^+^ LN and BCL6^+^ GC-T_FH_ cells were included in the analysis for assessment of GC-committed populations (**Figure 1B**). The induction of Env-specific T_FH_ cells in LNs was assessed by the Activation Induced Marker (AIM) assay using CD25 and OX40 (CD134) upregulation in T_FH_ cells stimulated with Env pooled peptides in comparison to unstimulated samples as previously described (42) (**Figure 1C**). Dynamics of total LN, GC and BCL6^+^ T_FH_ cells were monitored over the course of vaccination using LN biopsies collected pre-vaccination and at day 3 and day 14 post 2^nd^ Adeno and 2^nd^ boost. As animals biopsied at day 3 and at day 14 post-immunizations belonged to separate groups (see *Materials and Methods*) cellular frequencies were normalized to pre-immunization values and the fold-changes of frequencies obtained post-immunization are reported as magnitude of responses. Total LN T_FH_ cells were not significantly boosted over the course of immunization (**Figure 1D**) while GC-T_FH_ cells tended to increase at week 15, 2 weeks following the 2^nd^ Adeno immunization (**Figure 1E**). The frequencies of BCL6 expressing cells were significantly higher in GC-T_FH_ cells compared to total LN T_FH_ cells independent of the immunization timepoint (**Figure 1F**), confirming the GC program of PD-1^hi^ T_FH_ cells in LNs and induction of T_FH_ cell maturation (51, 52). BCL6^+^ GC-T_FH_ cells tended to increase at day 3 following the 2^nd^ adeno and significantly increased at day 3 following the 2^nd^ boost, indicating that the T_FH_ maturation was vaccine-dependent (**Figure 1G**). Env-specific LN T_FH_ cells were significantly induced at day 3 post-2^nd^ Adeno and exhibited higher levels at day 3 post-2^nd^ boost (**Figure 1H**). Further, the average frequency of Env-specific LN T_FH_ cells at week 38 + 3 days was 1.49%, 4.26 times higher in comparison to the average frequency observed in mock vaccinated animals (0.35%). Likewise, the magnitude of Env-specific GC-T_FH_ cells was significantly elevated at day 3 following the 2^nd^ boost (**Figure 1I**). The average frequency of Env-specific GC T_FH_ cells at week 38 + 3 days in immunized macaques was 2.9%, 4 times higher in comparison to that of the control-vaccine group (0.7%). Taken together these results indicate efficient elicitation of vaccine-dependent Env-specific T_FH_ responses in LNs of immunized rhesus macaques and that strong T_FH_ responses were particularly induced following the intramuscular protein boosts.

### Immunization Induces GC Expansion and Env-Specific B Cells in LNs

**Figure 2:**
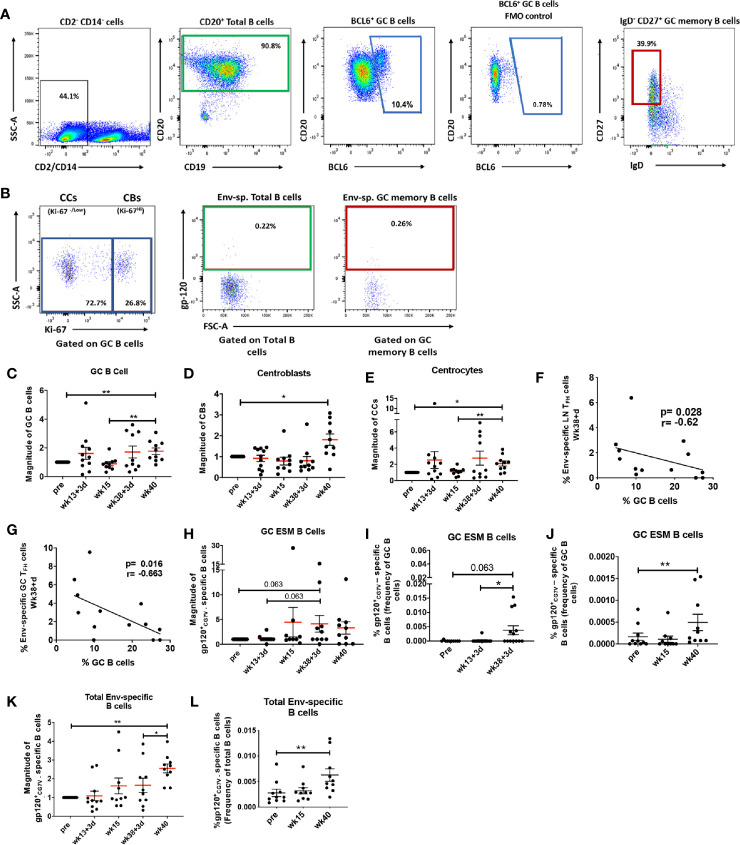
Vaccination induced GC B cell populations indicate efficient GC maturation. **(A)** Total B cells in LN were identified as CD20^+^ (green gate); GC B cells as BCL6^+^ B cells (blue gate) and GC memory B cells as IgD^-^ CD27^+^ GC B cells (red gate). **(B)** Centrocytes (CC) and Centroblasts (CB) were gated on GC B cells and defined respectively as Ki67^neg or dim^ and Ki67^hi^. Env-specific B cells were identified as gp120^+^ and gated either on total CD20^+^ B cells or GC memory B cells. Dynamics of **(C)** GC B cells, **(D)** centroblasts, and **(E)** centrocytes assessed at pre, 3 days and 2 weeks post-2^nd^ prime (week 13 + 3 days and week 15), and 3 days and 2 weeks post-2^nd^ boost (week 38 + 3 days and week 40) and represented as magnitude of response. Negative correlations were seen between **(F)** Env-specific LN T_FH_ and **(G)** Env-specific GC T_FH_ cells and GC B cells at week 38 + 3 days. **(H)** Magnitude of GC Env-specific memory B cells over the course of vaccination. Paired analysis of non-normalized GC Env-specific memory B cell frequencies were performed on samples collected at **(I)** pre- and day 3 following immunizations and **(J)** pre- and 2 weeks following immunizations. **(K)** Magnitude of total Env-specific B cell response over the course of vaccination. **(L)** Paired analysis of non-normalized total Env-specific B cell frequencies within animals that had LNs collected 2 weeks post-immunizations. Statistics were generated using the Wilcoxon signed rank test for animals from the same group **(I, J, L)** or the Mann-Whitney U test for animals from different groups **(C–E, H, K)**. Correlation analyses were performed using Spearman correlation **(F–G, M)**. * indicates P < 0.05, ** indicates p < 0.01.

Formation of GCs in secondary lymphoid organs is pivotal for generation of long-lived antibody-secreting PCs and antigen-specific memory B cells, which can provide protection upon antigenic re-exposure (53). We next explored the ability of the vaccine regimen to induce GC expansion and generation of Env-specific B cells. Total B cells were defined as CD2^neg^ CD14^neg^ CD20^+^ B cells (**Figure 2A**). The BCL6 transcription factor identifies a subpopulation responsible for differentiation of GC B cells into antigen-specific PCs and memory B cells and is the canonical marker defining GC B cells in both humans and rhesus macaques (26, 51, 54–57). Memory B cells in LNs of rhesus macaques have been previously defined as CD27^+^ IgD^-^ B cells (58–60). Therefore, in this study, GC B cells were defined as BCL6^+^ B cells and GC memory B cells were defined as BCL6^+^ IgD^neg^ CD27^+^ cells (**Figure 2A**). The fully formed GC is divided into light and dark zones. The light zone contains centrocytes (CCs), GC B cells with low rates of proliferation and defined as Ki-67^neg^/Low GC B cells (**Figure 2B**). The dark zone contains centroblasts (CBs), highly proliferative GC B cells defined as Ki-67^Hi^ GC B cells (**Figure 2B**). Extrafollicular T helper cells can support generation of antigen-specific B cells outside GCs (61–63). To evaluate out-of-GC Env-specific B cell development, we analyzed Env-specific B cells within the absolute pool of CD20^+^ B cells and characterized this population as total Env-specific B cells (**Figure 2B**). GC Env-specific memory (GC ESM) B cells, gated within the GC memory B cell population, were also evaluated (**Figure 2B**). The percentages of LN B cell subsets were normalized to pre-immunization levels and results are reported as the magnitude of response. GC B cells (**Figure 2C**), centroblasts (**Figure 2D**), and centrocytes (**Figure 2E**) all significantly increased at week 40, 2 weeks after the 2^nd^ boost immunization, indicating the vaccine regimen promoted robust GC maturation. Notably, frequencies of Env-specific LN T_FH_ cells (**Figure 2F**) and Env-specific GC-T_FH_ (**Figure 2G**) cells obtained at day 3 post-2^nd^ boost (week 38 + 3 days) inversely correlated with absolute GC B cell frequencies at the same timepoint. The current model of B cell affinity maturation in GCs suggests that discrimination between low and high affinity antigen-specific B cells is driven by competition for survival signals coming from antigen-specific T_FH_ cells and that low affinity B cells are induced to enter apoptosis (64). The negative correlations support the idea that antigen specific-T_FH_ cells may have played a role in shaping the affinity of antigen-specific B cells by inducing apoptosis of non-specific B cell populations. As GC-generated memory B cells show higher affinity levels for cognate antigen (65) we assessed the dynamics of GC ESM B cells, which tended to increase at day 3 post-2^nd^ boost (**Figure 2H**). Of note, the average frequency of GC ESM B cells in mock-vaccinated macaques was 0.0013% (frequency of GC B cells) following the 2^nd^ boost. When non-normalized percentages were compared by paired analysis between animals of the same group, a marginally significant increase in GC ESM B cells at day 3 post-2^nd^ boost was observed in comparison to pre-vaccine levels (**Figure 2I**). However, GC ESM B cells were clearly induced at later timepoints as shown by significantly increased frequencies at week 40 in animals with LN collections 14 days following the last immunizations (**Figure 2J**). Total Env-specific B cells progressively increased over the course of vaccination (**Figure 2K**) and significant induction of this population compared to pre-vaccination frequencies was observed by paired analysis in macaques with 14-day post-immunization collections (**Figure 2L**). The average frequency of total Env-specific B cells within macaques in the control group was 0.0014% (frequency of B cells) following week 38 immunization. Overall, non-specific B subpopulations associated with GC maturation and also GC-dependent and GC-independent Env-specific B cells were expanded after the 2^nd^ boost. Env-specific T_FH_ cells were increased as early as day 3 following the 2^nd^ boost suggesting that LN T_FH_ responses preceded and may have supported GC maturation and Env-specific B cell generation.

### Early Induced Env-Specific B Cells and Env-Specific T_FH_ Cells in LNs Correlate With Env-Specific Mucosal Immunity

**Figure 3:**
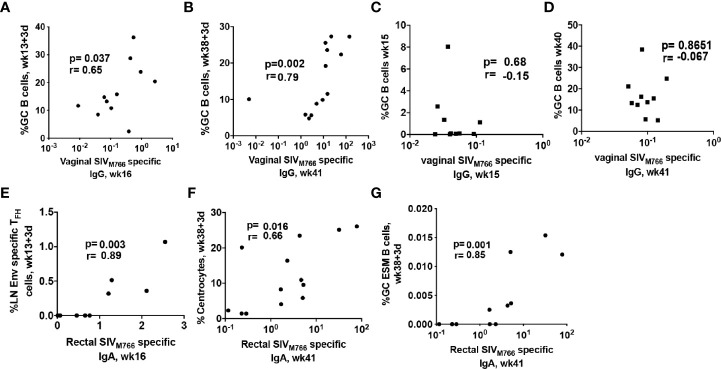
LN cellular subsets rapidly induced by immunization correlate with SIV-specific antibody responses at mucosal sites. Correlation between total GC B cells elicited at **(A)** day 3 post-2^nd^ adeno and vaginal Env-specific IgG responses at week 16 and **(B)** day 3 post-2^nd^ boost and vaginal Env-specific IgG responses at week 41. No correlation was seen between GC B cells elicited at **(C)** 2 weeks post-adeno and vaginal Env-specific IgG titers at week 16 or **(D)** GC B cells elicited at 2 weeks post-boost and vaginal Env-specific IgG titers at week 41. **(E)** LN Env-specific T_FH_ cells induced at day 3 post-2^nd^ adeno strongly correlated with Env-specific IgA titers in rectal secretions at week 16. Rectal Env-specific IgA antibody titers at week 41 positively correlated with B cell populations associated with GC maturation including **(F)** centrocytes and **(G)** GC Env-specific memory B cells. Correlation analyses were performed using Spearman correlations.

Generation of effective HIV-specific humoral immunity at rectal and vaginal mucosal sites is desirable for a protective HIV vaccine since most infections occur by the rectal/genital route (66). As functional mucosal IgG and IgA antibody responses are likely shaped within GC follicles, we investigated whether vaccine-induced GC responses contributed to induction of Env-specific antibodies in the genital tract of immunized female rhesus macaques. The absolute GC B cell population induced at day 3 following the 2^nd^-prime significantly correlated with Env-specific IgG antibodies in vaginal secretions at week 16, 3 weeks after the 2^nd^ prime (**Figure 3A**). Further, GC B cells at day 3 after the 2^nd^ boost correlated more strongly with Env-specific IgG responses in vaginal secretions at week 41, 3 weeks after the 2^nd^ boost (**Figure 3B**). In contrast, GC B cell frequencies at week 15 (**Figure 3C**) and week 40 (**Figure 3D**), 2 weeks post-2^nd^ Adeno and 2^nd^ boost respectively, did not correlate with vaginal Env-specific IgG responses, suggesting that GC B cells elicited at the earlier timepoint may have contributed more to generation of mucosal Env-specific humoral immunity. A previous study reported that T_FH_ cells in LNs of Peyer’s patches provoke high-affinity IgA secretion into the intestinal lumen (67). The frequency of LN Env-specific T_FH_ cells at day 3 following the 2^nd^ adeno displayed a strong correlation with Env-specific IgA antibodies elicited in rectal secretions at week 16, 3 weeks post-2^nd^ Adeno (**Figure 3E**), supporting the contribution of T_FH_ cells to selection of IgA antibodies in the intestinal mucosa. Centrocytes receive survival signals from T_FH_ cells and follicular dendritic cells (FDCs) in germinal centers, leading to their differentiation into antigen-specific PCs and memory B cells (68). Further analysis showed that GC centrocytes (**Figure 3F**) and GC ESM B cells (**Figure 3G**) induced as early as day 3 post-2^nd^ boost significantly associated with rectal Env-specific IgA antibodies elicited at week 41, confirming that efficient maturation of B cells in GCs also supported SIV-specific IgA antibody in rectal secretions. No correlations were seen between early induced T_FH_ cell and GC B cell responses in LN and a decreased rate of SIV acquisition following SIV vaginal challenge. Nevertheless, these findings support the essential contribution of early-induced Env-specific LN T_FH_ cells and Env-specific LN B cells to the generation Env-specific responses in mucosal tissues. Notably, these findings add robust evidence extending the results of our previous study (30), by showing clear associations of the Env-specific LN T_FH_ cells with Env-specific IgA antibodies in rectal secretions and Env-specific IgG antibodies in vaginal secretions with LN B cells.

### Vaccination Expands Env-Specific T_FH_ Cells in the Circulation of Immunized Rhesus Macaques

**Figure 4:**
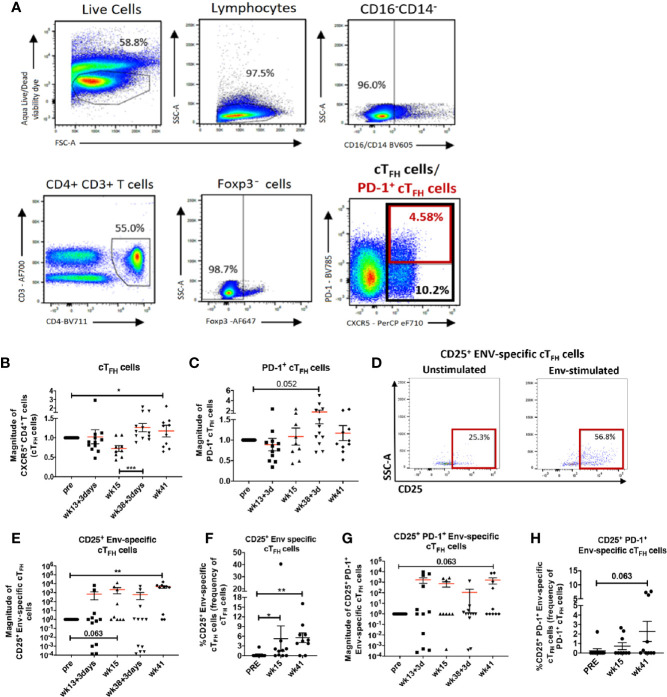
Vaccine induced cT_FH_ cells and Env-specific cT_FH_ cells in peripheral blood of immunized rhesus macaques. **(A)** gating strategy for identification of cT_FH_ (CXCR5^+^ CD4^+^ T cells) and PD-1^+^ cT_FH_ cells. Dynamics of **(B)** cT_FH_ cells and **(C)** PD-1^+^ cT_FH_ cells over the course of immunization represented as magnitude of response. **(D)** Identification of Env-specific cT_FH_ cells by flow cytometry using the activation-induced marker CD25. Circulating T_FH_ cells were either stimulated with Env-pooled peptides or remained unstimulated. **(E)** Dynamics of CD25^+^ Env-specific cT_FH_ cells over the course of immunization represented as magnitude of response and **(F)** paired analysis within animals that had blood collected at pre-vaccination, week 15 and week 41. **(G)** Dynamics of CD25^+^ PD-1^+^ Env-specific cT_FH_ cells over the course of immunization represented as magnitude of response and **(H)** paired analysis within animals that had blood collected at pre-vaccination, week 15 and week 41. Statistics were generated using the Wilcoxon signed rank test for animals within the same group **(B, C, E–H)** or the Mann-Whitney U test for animals from different groups **(B, C, E, G)**. * indicates P < 0.05, ** indicates p < 0.01.

Several studies have demonstrated that cT_FH_ cells play an important role in HIV-specific humoral immunity (15, 20, 22, 69). We next investigated whether immunization elicited cT_FH_ responses in the blood of female rhesus macaques and whether vaccine-induced cT_FH_ responses contributed to anti-HIV humoral immunity. There is general agreement that CXCR5^+^ CD4^+^ T cells in peripheral blood represent counterparts of LN T_FH_ cells (14, 70). In humans, cT_FH_ cells are heterogeneous and comprise distinct subpopulations of T helper cells (71, 72). For example PD-1^+^ cT_FH_ cells were reported to be more closely related to GC-T_FH_ cells and to possess GC-T_FH_ effector functions (73, 74). To reconcile our data with the mixed phenotypic definitions of cT_FH_ cells, we investigated both absolute circulating CXCR5^+^ CD4^+^ T cells, here defined as cT_FH_ cells, and PD-1^+^ cT_FH_ cell populations (**Figure 4A**). Blood collected for cT_FH_ cell analysis matched the same animal subgroups and timepoints as LN biopsies. Therefore, cT_FH_ cell levels were normalized to pre-vaccination values and reported as the magnitude of response. Both absolute cT_FH_ cells (**Figure 4B**) and PD-1^+^ cT_FH_ cells (**Figure 4C**) peaked at day 3 post-2^nd^ boost indicating that the Env protein immunization induced T_FH_ cell responses in peripheral blood along with LN T_FH_ responses.

Next, we evaluated induction of Env-specific cT_FH_ cells using a modified AIM assay as described in Methods and addressed further in the *Discussion* section. Env-specific cT_FH_ cells were defined as CD25^+^ Env-specific cT_FH_ cells (**Figure 4D**). The magnitude of CD25^+^ Env-specific cT_FH_ cells showed a marginally significant increase at week 15, 2 weeks following the 2^nd^ adeno, but a significant increase was seen at week 41, 3 weeks following the 2^nd^ boost (**Figure 4E**). Paired analysis of CD25^+^ Env-specific cT_FH_ cell frequencies at week 15 and week 41 showed significant elevations compared to the pre-immunization time point. (**Figure 4F**). We also detected a trend toward significance in elevated magnitude of PD-1^+^ CD25^+^ Env-specific cT_FH_ cells at week 41 in comparison to the pre-vaccination timepoint (**Figure 4G**), suggesting vaccine-dependent induction of Env-specific cT_FH_ cells with a GC-T_FH_ like phenotype. Further, a trend toward increased frequencies of PD-1^+^ Env-specific cT_FH_ cells at week 41 compared to pre-vaccine frequencies was also observed in paired analysis (**Figure 4H**). Taken together, our data show that the vaccine regimen induced both absolute cT_FH_ and PD-1^+^ cT_FH_ cells with expansion of CD25^+^ Env-specific cT_FH_ cell populations following the booster immunizations.

### Frequencies of cT_FH_ Cells Correlate With T_FH_ Cells in LNs

**Figure 5:**
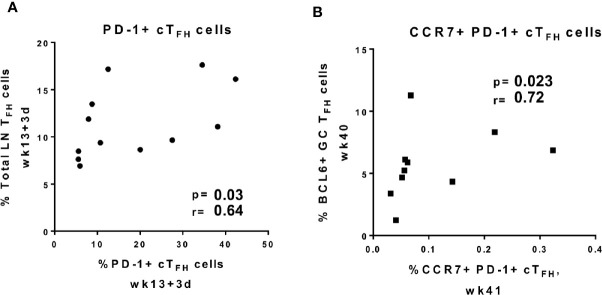
Circulating T_FH_ cell subpopulations positively correlate with GC T_FH_ cell subpopulations. Correlations between frequencies of **(A)** PD-1^+^ cT_FH_ cells elicited at day 3 post-2^nd^ adeno and total LN T_FH_ cells induced at same time point, **(B)** BCl6^+^ GC-T_FH_ cells induced at week 40, 2 weeks after the last boost, and CCR7^+^ PD-1^+^ cT_FH_ cells at week 41, 3 weeks after the last boost. Correlation analyses were performed using Spearman correlations.

Peripheral PD-1^+^ cT_FH_ cells with a central memory phenotype were suggested to originate from GC-T_FH_ cells in individuals immunized against influenza virus (75). Another study found that human cT_FH_ cells co-expressing CXCR5 and PD-1 exited LNs through the efferent flow of the thoracic duct (73). To further explore the origins of cT_FH_ cells in immunized rhesus macaques, we performed correlations between circulating T_FH_ cell subsets and LN T_FH_ cells following immunizations. Frequencies of absolute LN T_FH_ cells positively correlated with PD-1^+^ cT_FH_ cells at day 3 following the 2^nd^ adeno immunization (**Figure 5A**), suggesting PD-1^+^ cT_FH_ cells may originate from LN T_FH_ cells in rhesus macaques and that Env-specific cT_FH_ cell subsets may be shed from LNs upon antigenic re-exposure. Moreover, central memory CCR7^+^ PD-1^+^ cT_FH_ cells at week 41 exhibited an even stronger positive correlation at week 40 with BCL6^+^ GC-T_FH_ cells, an activated population (**Figure 5B**). Our results indicate that re-exposure to cognate antigen may lead to the appearance of cT_FH_ cells with a memory phenotype in the periphery and this peripheral memory population may arise from GC-T_FH_ cells.

### Vaccine-Induced cT_FH_ Cells Correlate With Systemic and Mucosal Env-Specific Humoral Responses

Circulating T_FH_ cells were previously shown to support antibody release by peripheral B cells during HIV infection (14, 17, 22). Therefore, we investigated relationships between cT_FH_ cell populations and systemic and mucosal humoral responses to determine whether vaccine-induced cT_FH_ cells supported Env-specific humoral immunity in the periphery and mucosa of the female macaques. As cT_FH_ cells are heterogeneous (72), we categorized them into distinct functional subpopulations (**Table 2**) and examined correlations with Env-specific IgG or IgA secreting plasmablasts (PBs) and PCs in bone marrow (BM), Env-specific IgG titers in vaginal secretions and Env-specific IgA titers in rectal secretions. CD95^+^ PD-1^+^ cT_FH_ cells induced at day 3 post-2^nd^ boost (week 38 + 3 days) presented a marginally significant correlation with Env-specific IgG secreting PBs/PCs in BM, while CCR6^+^ PD-1^+^cT_FH_ and CCR7^+^ PD-1^+^ cT_FH_ cells exhibited significant correlations suggesting that these memory cT_FH_ subsets induced promptly after boosting contributed to systemic Env-specific antibody responses. PD-1^+^ cT_FH_ cells, a phenotype closely associated with T_FH_ cells in LN (73), expressing CD95, CCR6 and CCR7 induced at wk38+3d also significantly correlated with Env-specific IgA responses in BM, indicating that these rapidly induced cT_FH_ memory subpopulations also supported IgA Env-specific immunity systemically.

**Table 2:** Immunological correlates between circulating T_FH_ cell subpopulations and vaccine-induced humoral responses in bone marrow and at mucosal sites.

Vaccine-induced Env-specific responses	cT_FH_ phenotype	cT_FH_ functional subpopulation	r value	P value
**Bone Marrow IgG-secreting PBs/PCs**	CD95^+^ PD-1^+^ cT_FH_	Total memory	0.58	0.052^§^
	CCR6^+^ PD-1^+^ cT_FH_	Th17-like memory	0.72	0.011
	CCR7^+^ PD-1^+^ cT_FH_	Central memory	0.67	0.019
**Bone Marrow IgA-secreting PBs/PCs**	CD95^+^ PD-1^+^ cT_FH_	Total memory	0.77	0.005
	CCR6^+^ PD-1^+^ cT_FH_	Th17-like memory	0.84	0.001
	CCR7^+^ PD-1^+^ cT_FH_	Central memory	0.59	0.047
**Vaginal IgG titers**	CD25^+^ PD-1^+^ cT_FH_	Effector memory	-0.66^#^	0.022
	CD95^+^ PD-1^+^ cT_FH_	Total memory	-0.66^#^	0.024
	CXCR3^-^ PD-1^+^ cT_FH_	Resting memory	-0.69^#^	0.015
**Rectal IgA titers**	Total cT_FH_		0.71	0.01
	CD95^+^ PD-1^+^ cT_FH_	Total memory	-0.57^#^	0.059^§^
**Rectal IgA titers**	Total cT_FH_**		0.93	<0.001
	PD-1^+^ cT_FH_**	GC-like cT_FH_ cells	0.65	0.049
	CD95^+^ PD-1^+^ cT_FH_**	Total memory	0.65	0.049
	CD25^+^ PD-1^+^ cT_FH_**	Effector memory	0.93	0.003
	CCR6^+^ PD-1^+^ cT_FH_**	Th17-like memory	0.65	0.049
	CXCR3^-^ PD-1^+^ cT_FH_**	Resting memory	0.66	0.044

All cT_FH_ frequencies were calculated based on the total CD4^+^ T cell population. Analyses were performed using Spearman’s rank correlation. PBs, plasmablasts; PCs, plasma cells. ^#^indicates negative correlations. ^§^indicates near significant correlations. Vaccine-induced Env-specific humoral responses in first column were assessed at week 41; cT_FH_ cell populations in second column were assessed at week 38 + 3 days with exception of cT_FH_ cell populations indicated by ** which were assessed at week 41.

However, correlation analysis between cT_FH_ populations and SIV-specific antibody titers in the mucosa presented mixed results. While PD-1^+^ cT_FH_ cells elicited at week 38 + 3 days showed positive correlations with SIV-specific IgG and IgA secreting B cells in bone marrow as described above, PD-1^+^ cT_FH_ cell populations induced at the same timepoint exhibited negative correlations with Env-specific IgG titers in vaginal secretions (**Table 2**), suggesting that early induced cT_FH_ cells may not have contributed to anti-SIV IgG responses in the vaginal mucosa. In addition, no association between cT_FH_ populations induced by the 2^nd^ protein boost and a decreased rate of SIV acquisition following vaginal challenge was seen. Although the frequency of CD95^+^PD-1^+^cT_FH_ cells induced at week 38 + 3 days showed a trend toward a significant negative correlation with Env-specific IgA titers in rectal tissue, frequencies of total cT_FH_ cells induced at the same time point exhibited a strong positive correlation with rectal IgA titers, suggesting a role for cT_FH_ cells in supporting rectal IgA responses. Further, PD-1^+^ T_FH_ subpopulations evaluated at week 41, a later timepoint, showed strong positive correlations with Env-specific IgA titers in rectal tissue. Whilst these results were surprising, they indicate that the use of cT_FH_ cells as biomarkers for vaccine-induced SIV-specific antibodies in the mucosa may depend on the timepoint of cT_FH_ cell evaluation and that cT_FH_ cells may associate differently with IgG and IgA responses in distinct mucosal compartments.

### Circulating T_FH_ Cells Support the Plasma Cell Phenotype and IgA Release *In Vitro*

**Figure 6:**
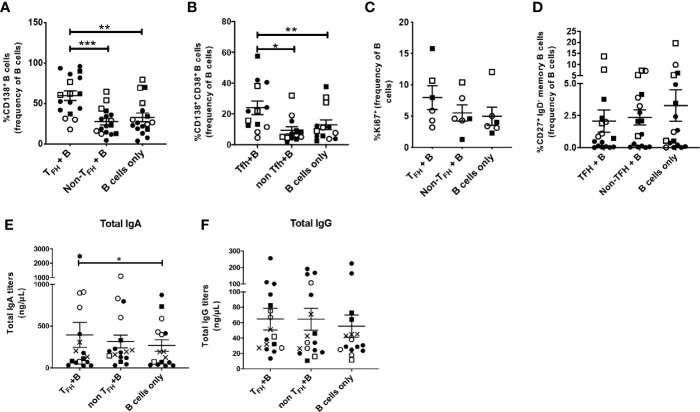
Circulating T_FH_ cells support plasma cell phenotype in co-cultured autologous B cells. CXCR5^+^ CD4^+^ cT_FH_ cells were isolated from blood samples of rhesus macaques obtained at necropsy and co-cultured with autologous enriched B cells. B cells were also co-cultured alone or in the presence of CXCR5^-^ CD4^+^ enriched T cells (non-cT_FH_ cells). Samples were included from both SIV-infected and protected animals for general assessment of T_FH_/B-cell help capacity with robust statistical power. B cells were analyzed by flow cytometry after 7 days of co-culture. CD38 expression could not be assessed in all T_FH_ co-cultured B cells while intracellular staining with anti-Ki67 could only be performed in samples with relatively high cell counts. Frequencies of **(A)** CD138^+^ B cells (n=17), **(B)** CD138^+^ CD38^+^ B cells (n=13), **(C)** Ki67^+^ B cells (n=6) and **(D)** CD27^+^ IgD^-^ memory B cells (n=17) were compared between cT_FH_+B cells, non-cT_FH_+B cells and B cells alone conditions. cT_FH_-dependent **(E)** IgA and **(F)** IgG responses were assessed in supernatants of the three different co-cultures by ELISA. Squares: protected animals; circles: chronically infected animals; solid symbols: vaccinated animals; open symbols: non-vaccinated animals; X: naïve animals. Statistical analysis was performed using the Wilcoxon Sign-rank test in all panels. * indicates P < 0.05, ** indicates p < 0.01, *** indicates < 0.001.

Previous studies reported that sorted peripheral T_FH_ cells of healthy adults supported maturation of autologous B cells into antibody secreting cells in coculture assays (22, 24). To further explore the B cell help provided by cT_FH_ cells in rhesus macaques, we magnetically sorted cognate CXCR5^+^ CD4^+^ cT_FH_ cells, CXCR5^neg^ CD4^+^ non-T_FH_ cells and CD19^+^ B cells from PBMCs and cultured B cells with cT_FH_ cells, non-cT_FH_ cells or B cells alone as described in Methods. We used PBMCs obtained at necropsy for cT_FH_ and B cell isolation, as blood collected over the course of immunization lacked the number of cells necessary for sorting and flow cytometry analysis. CD138 (syndecan-1) expression on B cells distinguishes circulating PCs and PBs from other functional B cell subsets. CD138 was significantly upregulated in B cells co-cultured with cT_FH_ cells compared to B cells co-cultured with non-cT_FH_ cells and B cells co-cultured alone (**Figure 6A**). Circulating PCs in rhesus macaques have been characterized by high expression of both CD38 and CD138 (76, 77). We observed induction of CD138^+^ CD38^+^ B cells when co-cultured with cT_FH_ cells, significantly more compared to B cells co-cultured with non-cT_FH_ and B cells alone (**Figure 6B**). We also assessed T-cell induced B cell expansion by Ki67 expression. Ki67^+^ B cells were increased 1.4-fold and 1.6-fold compared to B cells co-cultured with non-cT_FH_ and B cells alone, respectively (**Figure 6C**) although the increases were not statistically significant. Peripheral T cells producing IL-21 have been reported to promote differentiation of human memory B cells into PCs (78). Although we did not quantify IL-21 production in cT_FH_ cells co-cultured with B cells in this assay, IL-21 release has been previously demonstrated to be a hallmark of CXCR5^+^ cT_FH_ cells (79). Despite increased PC frequencies following culture of B cells with cT_FH_ cells (**Figure 6B**) we did not see a corresponding decline in the frequency of memory B cells (**Figure 6D**). We reasoned that the presence of cT_FH_ cells could be contributing to increased antibody production and analyzed co-culture supernatants for T_FH_-dependent antibody release. Co-culture supernatants from all animals, including chronically infected, protected and naïve macaques, exhibited increased IgA levels in supernatants from B cells co-cultured with cT_FH_ cells compared to B cells alone (**Figure 6E**). No significant differences were observed in IgG titers when the 3 culture conditions were compared (**Figure 6F**), suggesting that the stimulus provided by autologous cT_FH_ cells may have favored IgA antibody production.

### B-Cells Co-Cultured With cT_FH_ Cells Have Different Gene Expression Compared to B Cells Alone

To further explore the influence of cT_FH_ cells on B cells, PBMC samples were obtained from three naïve macaques for isolation of T_FH_ and B cell populations, as cell numbers of PBMCs collected post-immunization were too limited. Subsequently, B cells co-cultured with cT_FH_ cells or cultured alone were magnetically enriched and RNA was extracted to investigate gene expression by RT-PCR array. Of 84 genes included in the array, 20 were upregulated from 2-fold to more than 20-fold in B cells co-cultured with cT_FH_ cells compared to B cells cultured alone (**Table 3**). The upregulated genes included genes associated with chemotaxis, antibody production, proliferation, maturation and pro-inflammatory and anti-inflammatory cytokines. IL-7 and SPP1, related to proliferation, and CCL3, CXCL1, and CXCL5, involved in chemotaxis and migration, were among the most upregulated. Moreover, CD40L and IL-13, markers shown to prominently contribute to antibody production by B cells, were also upregulated. Taken together the results demonstrate a clear influence of cT_FH_ cells on regulation of B cell gene expression, in alignment with B cell phenotype analysis by flow cytometry.

**Table 3:** Cytokine and chemokine gene expression of B cells co-cultured with circulating T_FH_ cells compared to B cells alone.

Gene Category	Gene Symbol	Gene Title	Fold-Up regulation
**Antibody production**	**CD40LG**	CD40 ligand	**16.86**
	**IL-13**	Interleukin 13	**4.72**
**Anti-inflammatory cytokines**	**IL-2**	Interleukin 2	**16.88**
	**IL-24**	Interleukin 24	**1.99**
**Chemotaxis and migration**	**CCL3**	Chemokine (C-C motif) ligand 3	**>50**
	**CXCL1**	Chemokine (C-X-C motif) ligand 1	**>50**
	**CXCL5**	Chemokine (C-X-C motif) ligand 1	**>50**
	**CCL21**	Chemokine (C-C motif) ligand 21	**2.02**
	**CCL24**	Chemokine (C-C motif) ligand 24	**1.8**
	**CX3CL1**	Chemokine (C-X3-C motif) ligand 1, fractalkine	**1.6**
**Pro-inflammatory cytokines**	**IL-1α**	Interleukin 1 alpha	**50**
	**IL-17α**	Interleukin 17 alpha	**42.77**
	**IL-23α**	Interleukin 23 alpha	**9.75**
**Proliferation and differentiation**	**SPP1**	Secreted Phosphoprotein 1, Osteopontin	**>50**
	**GPI**	glucose phosphate isomerase, neuroleukin	**>50**
	**IL-7**	Interleukin 7	**>50**
	**IFN-γ**	Interferon gamma	**11.2**
	**TNFβ (LTA)**	Tumor necrosis factor beta	**9.8**
	**TNFSF10 (TRAIL)**	Tumor Necrosis Factor (Ligand) Superfamily, Member 10	**5.1**
	**IL-3**	Interleukine-3	**1.61**

Fold up-regulation is represented by ΔΔCT values. Bold values represent the fold up-regulation of gene expression of B cells co-cultured with circulating TFH cells compared to B cells alone, calculated by the ΔΔCT method.

### cT_FH_ Cells Show B Cell Help Capacity After SIV Infection

**Figure 7:**
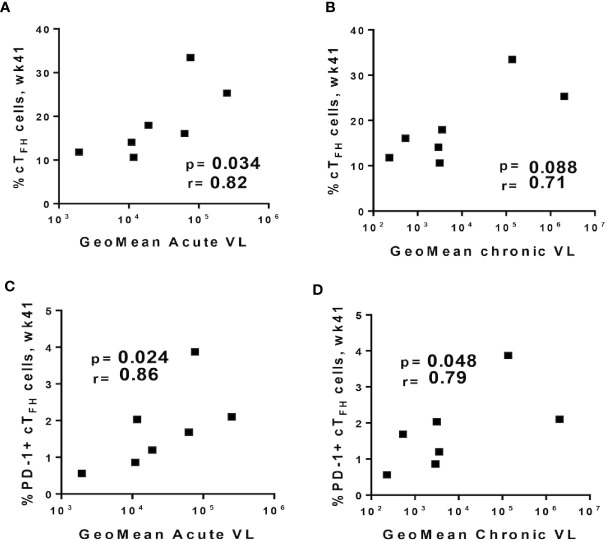
Relationship between circulating T_FH_ cell subpopulations prior to SIV challenge and plasma viral loads. Total cT_FH_ cells at week 41 were positively correlated with geometric means of **(A)** acute (weeks 1–6 post-infection) and **(B)** chronic (weeks 8–32 post-infection) viral loads. PD-1^+^ cT_FH_ cells at week 41 positively correlated with geometric means of **(C)** acute and **(D)** chronic viral loads. Correlation analyses were performed using Spearman correlations.

Despite the role of cT_FH_ cells in providing B-cell help in peripheral blood, some studies have indicated that cT_FH_ cells may also function as the main viral reservoirs during HIV infection (80, 81), prompting us to explore the possible contribution of cT_FH_ cells to SIV replication. We correlated frequencies of cT_FH_ subsets obtained at week 41, the latest timepoint prior to SIV repeated challenges, with acute (weeks 1–6 post-infection), and chronic plasma viral loads (weeks 8–32 post-infection) of infected macaques. A significant positive correlation was observed between the frequency of absolute cT_FH_ cells and acute viremia (**Figure 7A**), and a positive trend was seen with chronic viremia (**Figure 7B**). PD-1^+^ cT_FH_ cells, a subset closely related to the GC-T_FH_ phenotype, displayed strong positive correlations with both acute (**Figure 7C**) and chronic (**Figure 7D**) viral loads in agreement with studies suggesting that cT_FH_ cells contribute to viral replication (80, 82). Nevertheless, because cT_FH_-B cell interactions in blood have been associated with the quality of antibody responses in HIV^+^ individuals (8, 24), we investigated whether B cell help provided by cT_FH_ cells would differ between infected and uninfected animals. As described in Methods, cT_FH_-B cell co-culture experiments included necropsy samples from chronically infected (n=11) and protected animals (n=6) collected respectively at 40 weeks post-infection and 32 weeks following the last challenge. The chronic viremia of infected macaques differed. Some exhibited high viral loads (HVL, geomean > 10^4^ SIV RNA copies/ml) and some low viral loads (LVL, geomean ≤ 10^4^ SIV RNA copies/ml). However, B cells from HVL and LVL animals showed similar frequencies of PCs, memory B cells and Ki67^+^ B cells in all co-culture conditions. Therefore, data from both HVL and LVL groups were combined for comparison with protected macaques.

**Figure 8:**
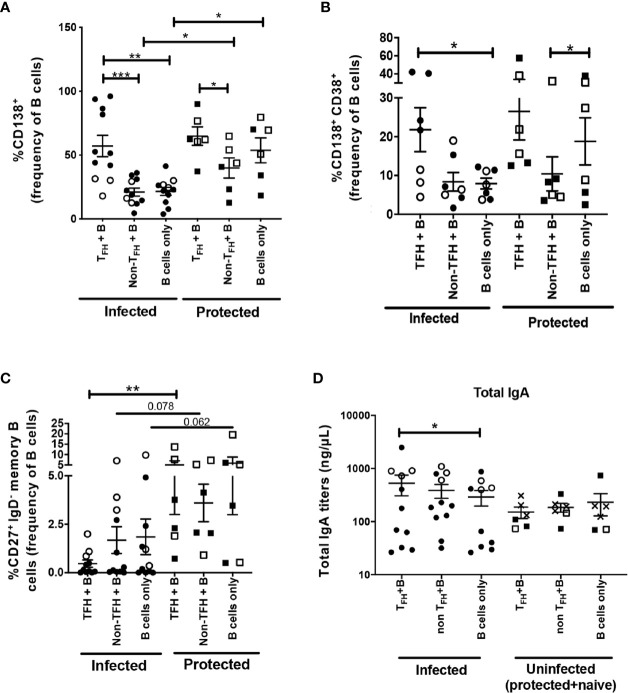
B-cell help capacity of cT_FH_ cells from chronically infected and uninfected rhesus macaques. Phenotypic comparison of B cells co-cultured with cT_FH_ cells (CXCR5^+^ CD4^+^ T cells), non-cT_FH_ cells (CXCR5^-^ CD4^+^ T cells) or cultured alone between chronically infected animals and protected animals. CD38 expression could not be assessed in all T_FH_ co-cultured B cells. B cell frequencies were assessed for **(A)** CD138^+^ B cells (n=17, 11 infected and 6 protected), **(B)** CD138^+^ CD38^+^ B cells (n=13, seven infected and six protected) and **(C)** CD27^+^ IgD^-^ memory B cells (n=17, 11 infected and six protected). **(D)** IgA release in supernatants of different co-cultures between infected and uninfected macaques. Squares: protected animals; circles: chronically infected animals; solid symbols: vaccinated animals; open symbols: non-vaccinated animals; X: naïve animals. Analyses comparing different groups of macaques used the Mann Whitney U test **(A–D)**. Analyses within the same group of macaques used the Wilcoxon Sign-rank test **(A–D)**. * indicates P < 0.05, ** indicates p < 0.01, *** indicates < 0.001.

B cells co-cultured with cT_FH_ cells exhibited similar frequencies of CD138 expressing B cells when infected animals were compared to protected animals (**Figure 8A**). Likewise, frequencies of CD38^+^CD138^+^ double positive B cells in cT_FH_ + B cell co-cultures of chronically infected animals were no different than co-cultures of protected animals (**Figure 8B**). These results support the notion that cT_FH_ cells contributed to the PC phenotype of autologous B cells in both chronically infected and protected macaques.

B cells co-cultured with cT_FH_ cells in infected animals presented significantly lower levels of CD27^+^ IgD^neg^ memory B cells in comparison to the protected animals (**Figure 8C**). This raises the possibility that cT_FH_ cells from infected macaques may have an enhanced capacity to promote memory B cell maturation into PCs. To address this hypothesis, cT_FH_ cells co-cultured with autologous B cells were assessed for IL-21 and BAFF expression, the principal T_FH_ cytokines involved in memory B cell differentiation into PC phenotype (83). Averages of IL-21^+^ and BAFF^+^ cT_FH_ frequencies from infected animals were higher compared to protected animals, but no statistical significance was observed when frequencies of both IL-21^+^ and BAFF^+^ cT_FH_ cells were compared between infected and protected macaques (**Figures S1A, B**), suggesting that alteration of cT_FH_ function during SIV infection is not the only factor contributing to decreased memory B cell responses in circulation of rhesus macaques. Of note, B cells cultured alone in infected animals also presented lower frequencies of CD27^+^ IgD^neg^ memory B cells compared to B cells alone in uninfected group, however with a trend toward significance (**Figure 8C**), indicating that chronic SIV infection may have impaired accumulation of memory B cells in the periphery regardless of cT_FH_ cell function. Whether cT_FH_ cells during chronic infection can differentially support memory B cell maturation into PCs during HIV or SIV infection requires further investigation. Unexpectedly, when samples included in our co-culture supernatant analysis were categorized into infected and uninfected macaque groups, we observed significantly higher IgA titers in cT_FH_-co-cultured B cell supernatant of infected macaques, but not in the supernatant of uninfected animals (**Figure 8D**), indicating that cT_FH_ cells from infected animals might possess a higher capacity to support IgA responses compared to cT_FH_ cells from uninfected animals. Possible IgA-skewed responses supported by cT_FH_ cells in the context of viral infection should be explored in future research.

## Discussion

The RV144 HIV vaccine trial stimulated great interest in design of new strategies to improve development and function of anti-HIV Env antibodies (84). While most vaccine studies have focused on the breadth, magnitude, specificity, or function of vaccine-induced antibody responses (4), relatively little attention has been given to underlying mechanisms that promote generation of high-affinity Env-specific antibodies (18). Functional cross-talk between T_FH_ cells and B cell populations in secondary lymphoid organs is critical for GC expansion and clonal selection of antigen-specific long-lived PCs and memory B cells with higher antibody specificity (85–88). Efforts to improve anti-Env humoral responses would benefit from exploration of vaccine-elicited T_FH_ cell responses and their B cell helper functions. Here we conducted a comprehensive investigation of the dynamics of LN T_FH_ cells and cT_FH_ cells over the course of a vaccine regimen, examining the role of these T helper cell populations in GC maturation and induction of Env-specific humoral immunity in the periphery and at mucosal sites.

Although the vaccine regimen investigated here did not induce a significant decrease in rate of SIV acquisition in female rhesus macaques (31) and no correlation between T_FH_ responses and SIV acquisition delay was observed in this study, we observed vaccine induction of GC-T_FH_ and cT_FH_ cells that contributed to elicitation of antibody responses. However, the T_FH_-dependent antibody responses investigated here were not potent or broad enough to contribute to significant protection, suggesting that an improved Env immunogen may be needed in future strategies. Early T_FH_ activation has also been associated with potent antibody responses in HIV infection and optimal GC reactivity (12, 87, 89). While our previous study reported the importance of early induction of GC-T_FH_ responses for efficient GC maturation in LN (30), here we extended those findings, uncovering an association between early SIV-specific T_FH_ cell induction in LN and development of SIV-specific antibody responses in mucosal compartments (**Figure 3**). We observed expansion of GC-T_FH_ cells with increased BCL6 expression, the master GC transcription factor (55, 56), at day 3 following immunization indicating efficient maturation of GC-T_FH_ cells (**Figure 1**), fundamental for generation of durable humoral responses against the HIV-gp120 protein (90). We also saw significant elevation of vaccine-induced Env-specific T_FH_ subsets as early as 3 days following the systemic boosts which contributed to GC B cell clonal selection, as suggested by the negative association with absolute GC B cells (**Figure 2**). Additionally, we detected significant expansion of CCs and CBs, hallmarks of mature GC formation. Their expansion following the peak T_FH_ response is suggestive of T_FH_-driven GC maturation promoted by the gp120 booster immunizations. Nevertheless, while higher T_FH_ responses with GC phenotype were obtained at day 3 after the boost, GC T_FH_ cell frequencies seemed to decline within 2 weeks. Studies suggested that once T_FH_ cells have differentiated into GC T_FH_ cells and provided help to GC B cells, they may downregulate BCL6 towards development of a memory phenotype and emigrate from GCs (86, 91). This hypothesis is supported by the lower frequencies of BCL6^+^ GC T_FH_ cells observed 2 weeks following the booster immunizations (**Figure 1G**).

Antigen-specific memory B cells and long-lived PCs derived from GCs are generated by re-iterative cycles of expansion in which B cells undergo Darwinian T_FH_-dependent selection leading to release of antibodies with improved antigenic affinity (92). Numerous studies have reported the contribution of mucosal antibodies to protection against SIV/SHIV challenge (66, 93–95). Here, early vaccine-induced GC B cell subsets were correlated with Env-specific humoral responses in the female rectal-genital tract, supporting the importance of early T_FH_-dependent B cell maturation for elicitation of Env-specific antibodies at mucosal sites. Overall, our current findings indicate that rapid priming of T cells and generation of Env-specific LN T_FH_ subsets may also lead to potent GC reactions and improved Env-specific mucosal B cell responses.

Similar to T_FH_ cells in LNs, both cT_FH_ cells and CD25^+^ Env-specific cT_FH_ cells significantly expanded in the blood following the 2^nd^ boost vaccination. For detection of Env-specific cT_FH_ cells, we modified the AIM assay using the activation induced marker CD25, but not CD134 (OX40). Previous studies have successfully used both activation markers for detection of rare antigen-specific T cells in circulation (42, 96, 97). Nevertheless, delayed CD134 upregulation has been reported following *in vitro* stimulation of cells (98). In HIV-infected patients, detection of Env-specific T cells in PBMCs by the CD25/CD134 AIM assay was rare (99), suggesting that CD134 may lack the sensitivity needed to detect very rare T_FH_ populations in the periphery. While we detected CD134^+^ cT_FH_ cells by flow cytometry, we did not observe a significant difference in CD134 expression when comparing Env-stimulated and non-stimulated conditions, indicating that delayed CD134 upregulation may have been a limiting factor for the AIM assay in PBMC samples.

Whether CXCR5^+^ CD4^+^ T cells are memory counterparts of LN T_FH_ cells in blood is still disputed. Here, induction of BCL6^+^ T_FH_ cells in GCs coincided with the expansion of absolute cT_FH_ cells and Env-specific cT_FH_ cells, which led us to investigate whether cT_FH_ cells originated from GC-T_FH_ populations. T_FH_ -like cells with the GC-T_FH_ genetic imprint have been found in the circulation of humans and rhesus macaques (18, 75, 100). Further, vaccine studies have suggested that immunization promotes expansion of GC-derived cT_FH_ cells in peripheral blood (75, 101). We found positive correlations of PD-1^+^ cT_FH_ cells and Env-specific cT_FH_ cells with LN-T_FH_ cells as early as day 3 following immunizations, supporting the notion that LN and circulating T_FH_ cellular populations are related. However, a significant trend in elevation of GC T_FH_ cells was seen at week 15, 2 weeks following the 2^nd^ Ad5-SIV administration, and a concurrent decrease of the cT_FH_ population was observed at the same timepoint (**Figures 1E** and **4B**). The opposite dynamics observed in GC T_FH_ and cT_FH_ populations may indicate that the Adeno immunization efficiently induced LN-T_FH_ commitment with the GC reaction, which might have diminished the shedding of LN-T_FH_ cells into peripheral compartments. Vella et al (73). recently demonstrated that the efferent circulation of lymph promotes trafficking of CXCR5^+^ PD-1^+^ T_FH_ cells into the blood stream through the thoracic duct and provided convincing evidence that cT_FH_ cells represent a re-circulating pool originating in LN GCs. Our correlative data are in agreement with these findings. We also observed a stronger correlation between CCR7^+^ PD-1^+^ cT_FH_ cells and GC-T_FH_ cells after the 2^nd^ booster immunization. In humans, circulating CCR7^+^ PD1^+^ CXCR5^+^ CD4^+^ T cells were shown to be antigen-experienced cT_FH_ cells able to return to nondraining secondary lymphoid organs to support rapid GC formation upon antigen reencounter (74). Likewise, generation of CCR7^+^ PD-1^+^ cT_FH_ cells, central memory cells by virtue of CCR7 expression (20, 69), may also play a role in faster GC reactions following antigen re-exposure in immunized rhesus macaques.

Our study revealed positive correlations between cT_FH_ cells with both IgG and IgA Env-specific PBs and PCs in bone marrow, suggesting that cT_FH_ cells may be beneficial for antibody responses systemically. Circulating T_FH_ cells have been continually suggested to promote production of bNAbs and improve the quality of HIV-specific antibody responses in seropositive patients and in HIV-vaccine studies (8, 17, 20, 23). In concert with recent studies, our results endorse the role of cT_FH_ cells in providing B cell help and generation of Env-specific antibody responses in systemic compartments of rhesus macaques. Nonetheless, among the memory cT_FH_ cell subpopulations evaluated here, PD-1^+^ cT_FH_ cells, but not absolute cT_FH_ cells, correlated with systemic Env-specific B cells in bone marrow indicating that the B cell help capacity of CXCR5^+^ CD4^+^ T cells in the periphery is attributable to the PD-1^+^ cT_FH_ cell subset as previously proposed by others (8, 20, 22, 102). In this study, we also demonstrated that memory cT_FH_ cellular subsets presented mixed correlations with rectal Env-specific IgA titers and vaginal Env-specific IgG titers as shown in **Table 2**. While the negative correlations between early induced cT_FH_ cells and vaginal Env-specific IgG responses were unexpected, we showed consistent positive associations between PD-1^+^ cT_FH_ populations and IgA responses in rectal secretions. A recent report suggested that oral vaccination with Enterotoxigenic Escherichia Coli (ETEC) ETVAX in adult subjects elicited activated memory cT_FH_ responses associated with vaccine-specific IgA secreting intestinal PBs (103). As PD-1 expression characterizes the activated memory phenotype, we hypothesize that activated memory T_FH_ cells may have been mobilized into peripheral blood following termination of GC reactions in secondary lymphoid organs (**Figure 5**). Correlations between late PD-1^+^ cT_FH_ cells and Env-specific rectal IgA titers may reflect T_FH_ activity in LNs which contributed to the development of IgA secreting Env-specific memory B cells. Future studies should address this possibility. Collectively our results provide evidence that later accumulation of memory cT_FH_ cell subsets in blood may represent a biomarker associated with development of rectal SIV-specific IgA antibodies. Nonetheless, underlying mechanisms by which cT_FH_ cells contribute to elicitation of IgA Env-specific humoral responses in the rectal mucosa remain to be investigated in future studies.

To further address the contribution of cT_FH_ cells to B cell maturation we conducted co-culture experiments. An increased percentage of B cells with the PC phenotype was seen when peripheral B cells were cultured with autologous cT_FH_ cells, confirming the hypothesis that cT_FH_ cells support antibody responses by promoting B cell maturation into PCs. However, we also observed positive correlations between cT_FH_ cells induced following the latest boost and viremia following infection (**Figure 7**). Although our data suggest a possible contribution of T_FH_ cells to viral replication, the vaccine regimen and immunogens used here have been shown to elicit non-neutralizing antibodies and tier-1 SIV neutralization, but not neutralizing activity against the SIV_mac251_ challenge virus (38). Therefore, any transmitted virus, however limited, could have targeted susceptible T_FH_ cells. T_FH_ cells represent a major factor underlying the development of HIV specific bNAbs (104), believed necessary for protective immunity against HIV. Future vaccines that include an envelope immunogen designed to induce antibody breadth could take advantage of the enhanced help provided by vaccine-induced T_FH_ cells, leading to elicitation of potent bNAbs and prevention of infection, thus avoiding any exposure of susceptible T_FH_ cells to transmitted virus.

Circulating T_FH_ cells were previously shown to directly contribute to B-cell proliferation and differentiation into IgG and IgA producing PBs in human samples (14, 17, 24, 103). Here we observed skewed IgA release by B cells in the presence of cT_FH_ cells. Biased IgA responses were observed in supernatants of cT_FH_ co-cultured B cells of chronically infected animals, an effect not seen in uninfected animals. A previous study reported that IgA responses were slightly elevated in serum of HIV-infected patients compared to healthy donors (105) and increased IgA release in the supernatant of cT_FH_ and B cell co-cultures was detected using human PBMCs (103). While increased cytokine release by T_FH_ cells has been linked to IgA responses in mice (106), SIV replication has been suggested to promote accumulation of activated IL-21 producing T_FH_ cells with skewed B cell maturation in rhesus macaques (107). Other studies have shown that SIV infection in rhesus macaques and HIV infection in humans provokes Th1 polarization of T_FH_ cells (43, 108) while Th1 skewed T_FH_ cells were shown to enhance the magnitude of anti-SIV IgA responses (109). It is possible that SIV infection may have determined functional and phenotypic changes of cT_FH_ cells in blood which could have contributed to IgA-skewed release by peripheral B cells. Whether SIV infection can affect T_FH_-dependent IgA or IgG responses in the circulation of rhesus macaques remains to be elucidated in future studies.

Our gene expression data of B-cells co-cultured with cT_FH_ cells compared to B cells cultured alone revealed upregulation of cytokines and chemokines tightly associated with B cell activation, BCR engagement, B cell expansion, immunoglobulin rearrangement and antibody production. Taken together, the phenotypic and genetic analysis of B cells co-cultured with autologous cT_FH_ cells illustrate a potential mechanism for the B-cell help provided by T_FH_ cells in the blood of rhesus macaques, showing that cT_FH_ cells are able to support antibody production by shaping the genetic program of B cells into activated PCs.

In conclusion our findings highlight the importance of efficient T_FH_ cell induction in both secondary lymphoid organs and in the circulation for development of robust and durable specific humoral immunity against the HIV/SIV envelope in blood and in the female rectogenital tract. Therefore, strategies targeting enhanced T_FH_ responses are warranted for the success of future pre-clinical and clinical prophylactic vaccine trials.

## Data Availability Statement

The original contributions presented in the study are included in the article/**Supplementary Material**. Further inquiries can be directed to the corresponding author.

## Ethics Statement

The animal study was reviewed and approved by NCI Animal Care and Use Committee.

## Author Contributions

SH and MR-G: Conceptualization. SH, CH, MR, RH, ZM, and MR-G: Methodology. SH, CH, MR, RH, and MR-G: Investigation. SH, CH, MR, RH, ZM, and TH: Resources. SH: Writing—original draft. SH, CH, MR, RH, ZM, TH, and MR-G: Writing—review and editing. MR-G: Supervision. All authors contributed to the article and approved the submitted version.

## Funding

This work was funded by the Intramural Research Program of the National Institutes of Health, National Cancer Institute.

## Conflict of Interest

The authors declare that the research was conducted in the absence of any commercial or financial relationships that could be construed as a potential conflict of interest.

## Supplementary Material

Supplementary Figure 1 B-cell help capacity of cT_FH_ cells co-cultured with B cells. The B-cell help markers IL-21 and BAFF were assessed by intracellular staining and flow cytometry in CXCR5^+^ CD4^+^ cT_FH_ cells co-cultured with autologous B cells from chronically infected macaques (n=6) and protected macaques (n=6) at necropsy. Comparison of **(A)** IL-21^+^ cT_FH_ cells and **(B)** BAFF^+^ cT_FH_ cells in infected and protected animals. Statistical analysis was performed using the Mann-Whitney U test in both panels.
