# Supplementary figures and images for: TFH Cells Induced by Vaccination and Following SIV Challenge Support Env-Specific Humoral Immunity in the Rectal-Genital Tract and Circulation of Female Rhesus Macaques
Source: Front Immunol. 2021 Jan 28;11:608003. doi: 10.3389/fimmu.2020.608003 (PMC7876074; doi:10.3389/fimmu.2020.608003)

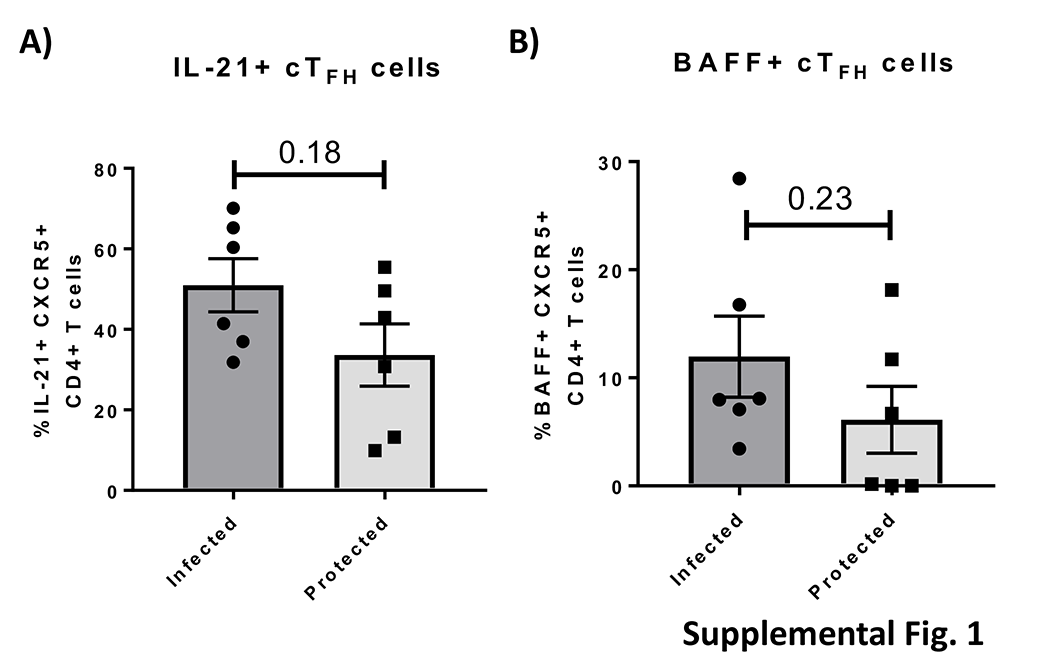

Supplement: Supplementary Figure 1 — B-cell help capacity of cTFH cells co-cultured with B cells. The B-cell help markers IL-21 and BAFF were assessed by intracellular staining and flow cytometry in CXCR5+ CD4+ cTFH cells co-cultured with autologous B cells from chronically infected macaques (n=6) and protected macaques (n=6) at necropsy. Comparison of (A) IL-21+ cTFH cells and (B) BAFF+ cTFH cells in infected and protected animals. Statistical analysis was performed using the Mann-Whitney U test in both panels. [file Image_1.tif]
